# Increases prognostic value of clinical-pathological nomogram in patients with esophageal squamous cell carcinoma

**DOI:** 10.3389/fonc.2023.997776

**Published:** 2023-02-14

**Authors:** Jing Feng Hu, Xin Song, Kan Zhong, Xue Ke Zhao, Fu You Zhou, Rui Hua Xu, Ji Lin Li, Xian Zeng Wang, Xue Min Li, Pan Pan Wang, Ling Ling Lei, Meng Xia Wei, Ran Wang, Zong Min Fan, Xue Na Han, Yao Chen, Liu Yu Li, Jia Jia Ji, Yuan Ze Yang, Bei Li, Miao Miao Yang, Hai Jun Yang, Fu Bao Chang, Jing Li Ren, Sheng Li Zhou, Li Dong Wang

**Affiliations:** ^1^ State Key Laboratory of Esophageal Cancer Prevention & Treatment of The First Affiliated Hospital, Zhengzhou University, Zhengzhou, Henan, China; ^2^ Henan Key Laboratory for Esophageal Cancer Research of The First Affiliated Hospital, Zhengzhou University, Zhengzhou, Henan, China; ^3^ Department of Thoracic Surgery, Anyang Tumor Hospital, Anyang, Henan, China; ^4^ Department of Pathology, Linzhou Esophageal Cancer Hospital, Linzhou, Henan, China; ^5^ Department of Thoracic Surgery, Linzhou People’s Hospital, Linzhou, Henan, China; ^6^ Department of Pathology, Hebei Provincial Cixian People’s Hospital, Cixian, Hebei, China; ^7^ Department of Surgery, Central Hospital of Linzhou City, Linzhou, Henan, China; ^8^ Department of Pathology, The Second Affiliated Hospital of Zhengzhou University, Zhengzhou, Henan, China; ^9^ Department of Pathology, Henan Provincial People’s Hospital, People’s Hospital of Zhengzhou University, People’s Hospital of Henan University, Zhengzhou, Henan, China

**Keywords:** esophageal squamous cell carcinoma, tumor-stroma ratio, nomogram, prognosis, overall survival

## Abstract

**Background:**

This study was intended to construct a brand new prognostic nomogram after combine clinical and pathological characteristics to increases prognostic value in patients with esophageal squamous cell carcinoma.

**Methods:**

A total of 1,634 patients were included. Subsequently, the tumor tissues of all patients were prepared into tissue microarrays. AIPATHWELL software was employed to explore tissue microarrays and calculate the tumor-stroma ratio. X-tile was adopted to find the optimal cut-off value. Univariate and multivariate Cox analyses were used to screen out remarkable characteristics for constructing the nomogram in the total populations. A novel prognostic nomogram with clinical and pathological characteristics was constructed on the basis of the training cohort (n=1,144). What’s more performance was validated in the validation cohort (n=490). Clinical-pathological nomogram were assessed by concordance index, time-dependent receiver operating characteristic, calibration curve and decision curve analysis.

**Results:**

The patients can divide into two groups with cut-off value of 69.78 for the tumor-stroma ratio. It is noteworthy that the survival difference was noticeable (*P*<0.001). A clinical-pathological nomogram was constructed by combining clinical and pathological characteristics to predict the overall survival. In comparison with TNM stage, the concordance index and time-dependent receiver operating characteristic of the clinical-pathological nomogram showed better predictive value (*P*<0.001). High quality of calibration plots in overall survival was noticed. As demonstrated by the decision curve analysis, the nomogram has better value than the TNM stage.

**Conclusions:**

As evidently revealed by the research findings, tumor-stroma ratio is an independent prognostic factor in patients with esophageal squamous cell carcinoma. The clinical-pathological nomogram has an incremental value compared TNM stage in predicting overall survival.

## 1 Introduction

Esophageal cancer (EC) is a highly malignant tumor with a poor prognosis. Apart from that, the 5-year survival rate of the EC patients with advanced stage is nothing more than about 10% (1). The morbidity and mortality of EC are considerably high in China. Worse still, about 200,000 people die of EC every year (2). The principal pathological types of EC are two types of esophageal squamous cell carcinoma (ESCC) and esophageal adenocarcinoma (EAC). ESCC is the predominant pathological type with 90% of the EC in China (3). So far, the determination of the prognosis of patients with ESCC primarily rests with the TNM stage, but the current research shows that the TNM staging method cannot predict the prognosis of patients well (4). What’s more, the accuracy of the prognosis of patients can be heightened by adding clinical and pathological factors (5, 6). As a result, new indicators need to be explored to evaluate the prognosis of patients.

As persuasively demonstrated by a multitude studies (7–9), the interstitial components of tumor tissue affect the prognosis of tumor patients. Nevertheless, owing to the lack of accurate and reliable evaluation methods for tumor stroma, the association between tumor stroma and patient prognosis is still unclear. This retrospective study explored the correlation between the tumor stroma and the overall survival (OS) as well as clinical characteristics of patients with ESCC by detecting the tumor-stromal ratio (the ratio of the area of stromal to the cancerous, TSR), to establish a classification criteria for TSR and to propose a brand new prognostic indicator. Aside from that, this study combined the TSR with the clinical and pathological characteristics of patients to construct a clinical-pathological nomogram, aiming to provide a better prediction model for the survival of ESCC.

## 2 Materials and methods

### 2.1 Study populations and follow-up

This study included 1,634 patients with pathologically diagnosed ESCC who were treated with radical surgery. The information of these patients comes from the tissue bank, clinical diagnosis, treatment, pathology, follow-up information database of about 500,000 cases of esophageal and gastric cardia carcinoma (1973-2021), established by the State Key Laboratory of Esophageal Cancer Prevention & Treatment and Henan Key Laboratory for Esophageal Cancer Research of The First Affiliated Hospital, Zhengzhou University. The clinical information of patients collected from the database, including gender, age at diagnosis, smoking history, drinking history, family history, high and low incidence area [the mortality, and mortality of esophageal cancer greater than 50/100,000 is defined as high incidence area (10)], and pathological information, including the degree of differentiation, T stage, N stage, M stage, as well as the patient’s H&E tissue microarrays. All patients were staged in accordance with to the 2002 American Joint Committee on Cancer (AJCC) esophageal cancer staging system. The documentation of informed consent was waived owing to anonymity of the participants. This study was approved by the Ethical Committee of The First Affiliated Hospital of Zhengzhou University. All procedures involving human participants were performed in accordance with the ethical committee’s standards and the Helsinki declaration.

Inclusion criteria: 1. Detailed and completed clinical data; 2. Received radical surgery for ESCC; 3. Postoperative pathological diagnosis was ESCC; Exclusion criteria: 1. Received radiotherapy or (and) chemotherapy before surgery; 2. Patients with distant metastasis; 3. Patients with other malignant tumors; 4. Patients who died within 30 days after surgery.

The follow-up of this study is principally through letters, telephone calls, home visits, and direct contact between village doctors and patients/their families, or through the new cooperative medical database, the medical security bureau database, and citizen death information registration management systems. It is carried out in an annual follow-up to record the time of death and the predominant cause of death to January 27, 2022. The primary clinical outcome was overall survival (OS).

### 2.2 Detection of tumor-stroma ratio

Tissue microarrays (TMAs) is a brand new method for high-throughput analysis of diagnostic or predictive markers in cancer specimens (11). What’s more, it has excellent applications in immunohistochemical analysis and observation of pathological features (12, 13). A total of 1,634 samples were histologically screened by H&E staining, and the sections were observed under a microscope. The 40 magnification was adopted to find the most infiltrating area of tumor tissue, and the representative area was marked in the paraffin block. Two cores (a cylinder with a diameter of 1.5 mm and a height of 3-5 mm) were taken out from the paraffin block to prepare a tissue chip. Each TMAs contains 150 cores (TMAs were constructed by Wuhan Servicebio Technology Co., Wuhan, China).

AIPATHWELL software (developed by Wuhan Servicebio Technology Co.) was utilized to scan and probe deep into the tissue microarray. The software distinguishes cancerous and stroma in accordance with tissue characteristics such as cell morphology and nucleus morphology. Apart from that, the output results are data such as cancerous area, stroma area. The tumor-stroma ratio (TSR) was expressed as the area of stroma/total area (stroma area + cancerous area). To ensure the accuracy of the results, we selected two cores for each patient and placed them on the tissue microarray. Subsequently, we took the mean as the final TSR value. The X-tile software was adopted to determine the cut-off value for the TSR. The X-tile software provides a convenient and comprehensive method to stratify the population by finding the optimal cut-off value based upon the patient’s overall survival time, when the difference in survival between these two groups is most striking (14).

### 2.3 Construction of the nomogram

The clinical and pathological information of patients were first considered as candidate variables for the prognostic model. It is noteworthy that all variables are categorical variables. The factors which affecting OS in univariate analysis (*P* < 0.05) were then included in Cox analyses. Subsequently independent prognostic risk factors (*P* < 0.05) were included as the final variables to construct the nomogram model. In the training set data, these characteristics were used to construct nomogram to predict the probability of patient survival time up to 1-year, 3-year, and 5-year by employing the rms package.

### 2.4 Calibration, validation and risk stratification of nomogram

Predictive performance was quantified with reference to accuracy (calibration), discrimination, and clinical application. The agreement between the predicted survival probability calculated by the nomogram and the patient’s actual survival probability was assessed by plotting 1-, 3-, and 5-year calibration curves for a bootstrapped with 1,000 sample. The discrimination of the nomogram model was assessed by calculating the concordance index (C-index), NRI and time-dependent receiver operating characteristic (ROC) (15). The clinical application and net benefit at diverse threshold probabilities of the nomogram model was assessed by decision curve analysis (DCA) (16). Patients in the study were divided into high-risk or low-risk groups in accordance with the optimal cutoff point for the total prognostic score (TPS) decided by the X-Tile software. Survival curves were drawn by adopting Kaplan-Meier analysis. The potential correlations between risk scores and OS among dissimilar subgroups were investigated in the overall patient population (14).

### 2.5 Statistical analysis

All statistical analyses were performed by employing the R software (version 4.2.0). T test was adopted to probe into continuous variables, categorical variables by adopting χ2 test and Fisher’s exact two-sided test. Survival analysis was drawn by employing Cox analysis and the Kaplan-Meier method. The nomogram and calibration plot were constructed using the “rms” package; the time-dependent receiver operating characteristic (ROC) was established by adopting the “timeROC” package; the DCA was established by employing the “rmda” and “ggDCA” package. *P* < 0.05 (two-sided) was considered statistically significant.

## 3 Results

### 3.1 Patient characteristics

The characteristics information of the 1,634 patients is shown in **Table 1**. The male to female ratio is 1.9:1; the mean age is 61 years, and the age range is 33-85. The median survival time of patients with ESCC was 2.0 years, and the 1-, 3- and 5-years OS rates were 77.2%, 35.1% and 23.7% respectively.

**Table 1:** Clinical characteristics of patients with esophageal squamous cell carcinoma.

Characteristics	Case (%)
Gender	
Male	1061 (64.93)
Female	573 (35.07)
Age at diagnosis in year	
Mean ± SD	61 ± 8
Smoking history	
Yes	781 (47.80)
No	853 (52.20)
Drinking history	
Yes	527 (32.25)
No	1107 (67.75)
Family history	
Yes	898 (54.96)
No	736 (45.04)
Area	
High incidence area	1138 (69.65)
Low incidence area	496 (30.35)
Tumor location	
Upper	271 (16.59)
Middle	1110 (67.93)
Lower	253 (15.48)
Differentiation	
Well	134 (8.20)
Moderate	975 (59.67)
Poor	525 (32.13)
T stage	
T1	55 (3.37)
T2	375 (22.95)
T3	1177 (72.03)
T4	27 (1.65)
N stage	
N1	832 (50.92)
N0	802 (49.08)

Patients were randomly divided into training and validation sets in a ratio of 7:3. The male to female ratio in the training set is 1.9:1; the mean age is 62 years, and the age range is 33-85. The median survival time of patients with ESCC was 2.0 years, and the 1-, 3- and 5-years OS rates were 76.5%, 35.1% and 24.1% respectively. The ratio of male to female in the validation set is 1.9:1; the average age is 61 years, and the age range is 38-82. The median survival time of patients with ESCC was 2.0 years, and the 1-, 3- and 5-years OS rates were 77.6%, 34.9% and 22.9% respectively.

### 3.2 Association analysis of TSR and clinical characteristics

**Figure 1:**
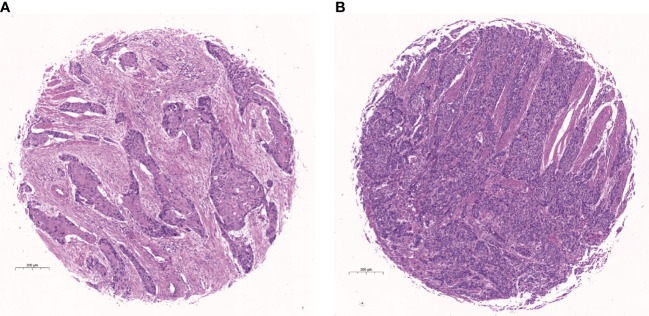
Hematoxylin-eosin (H&E) stained picture of the core of tissue microarray. **(A)** Example of stroma-rich group. **(B)** Example of stroma-poor group.

**Figure 2:**
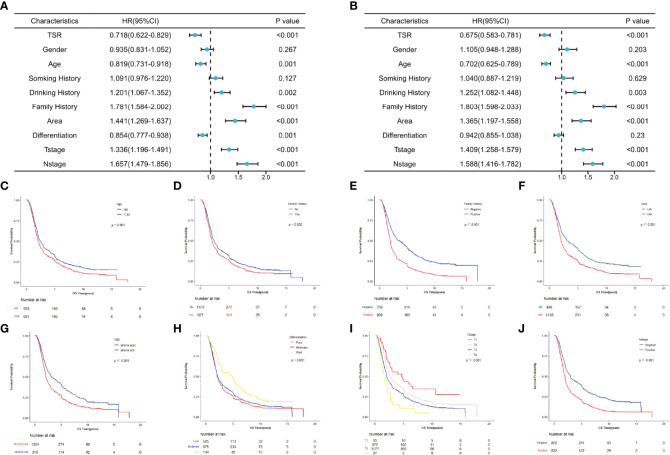
Survival analysis for patients with esophageal squamous cell carcinoma in overall populations. **(A)** Univariate analysis. **(B)** Multivariate analysis. **(C–J)** Survival curves for patients with age, drinking history, family history, area, TSR, differentiation, T stage and N stage, separately.

All patients successfully collected the detection value of TSR. The classification criteria for TSR were explored by comparing the relationship between TSR and survival for all patients. And the optimal cut-off value will be found when the survival difference was greatest. The optimal cut-off value was found by X-tile software. The result showed that patients were divided into two groups when the TSR cutoff value was 69.78, at which time the survival difference between the two groups was the greatest (P<0.001), and 69.78 was the optimal cutoff value for TSR. According to the optimal cut-off value, patients with TSR more than 69.78 were divided into stroma-rich group and patients with TSR less than 69.78 were divided into stroma-poor group (**Figure 1**). And a total of 1324 patients were divided into stroma-poor group and 310 patients were divided into stroma-rich group. The results showed that the abundance of the stroma in tumor tissue significantly affects the prognosis of patients, and patients survived significantly better when the stroma was abundant, and the TSR is an independent prognostic factor in patients with ESCC. The survival curves of the two groups are shown in **Figure 2G**. We applied the χ2 test to assess associations between TSR and clinical characteristics. We found that the TSR was significantly associated with the differentiation (*P* = 0.004) and depth of invasion (*P* = 0.002). In the stroma-rich group, the proportion of well-differentiated patients was relatively high, while, patients in the stroma-rich group had a relatively higher depth of infiltration. The relationship between TSR and differentiation and T stage may be the reason why it affects the prognosis of patients. The relationship between the clinical characteristics and the TSR is shown in **Table 2**.

**Table 2:** Relationship between TSR and clinical characteristics, n (%).

Characteristics	No. of The Patients Examined	Stroma Rich	Stroma Poor	P	OR	CI (95%)
Gender				0.375	0.888	0.683-1.154
Male	1061 (64.93)	208 (67.09)	853 (64.43)			
Female	573 (35.07)	102 (32.91)	471 (35.57)			
Age				0.98	0.997	0.886-1.281
≥60	953 (58.32)	181 (58.39)	772 (58.31)			
<60	681 (41.68)	129 (41.61)	552 (41.69)			
Area				0.501	0.913	0.699-1.191
High incidence area	1138 (69.65)	211 (68.06)	927 (70.01)			
Low incidence area	496 (30.35)	99 (31.94)	397 (29.98)			
Smoking History				0.983	0.997	0.779-1.277
Yes	781 (47.80)	148 (47.74)	633 (47.81)			
No	853 (52.20)	162 (52.26)	691 (52.19)			
Drinking History				0.684	1.056	0.812-1.374
Yes	527 (32.25)	105 (33.23)	424 (32.02)			
No	1107 (67.75)	207 (66.77)	900 (67.98)			
Family History				0.963	0.994	0.775-1.275
Positive	898 (54.96)	170 (54.84)	728 (54.98)			
Negative	736 (45.04)	140 (45.16)	596 (45.02)			
Location				0.184	–	–
Upper	271 (16.59)	50 (16.13)	221 (16.69)			
Middle	1110 (67.93)	222 (71.61)	888 (67.07)			
Lower	253 (15.48)	38 (12.26)	215 (16.24)			
Differentiation				0.004	–	–
Poor	525 (32.13)	81 (26.13)	444 (33.53)			
Moderate	975 (59.67)	192 (61.93)	783 (59.14)			
Well	134 (8.20)	37 (11.94)	97 (7.33)			
T stage				0.002	–	–
T1	55 (3.37)	10 (3.23)	45 (3.40)			
T2	375 (22.95)	46 (14.84)	329 (24.85)			
T3	1177 (72.03)	248 (80.00)	929 (70.16)			
T4	27 (1.65)	6 (1.93)	21 (1.59)			
N stage				0.984	1.002	0.783-1.284
N0	802 (49.08)	152 (49.03)	650 (49.09)			
N1	832 (50.92)	158 (50.97)	674 (50.91)			

### 3.3 Prognostic roles of clinical and pathological characteristics

To evaluate the potential association of clinical factors with overall survival, we performed univariate and multivariate Cox analysis in the total population, the prognostic role of each characteristic in OS was tested in the overall populations (**Figure 2**). And the univariate analysis results showed that age, drinking history, family history, area, TSR, differentiation, T stage and N stage were significantly associated with OS (*P* < 0.05) (**Figure 2A**), and multivariate Cox analysis results showed that age, drinking history, family history, area, TSR, T stage and N stage were independent predictors of OS (*P* < 0.05) (**Figure 2B**). Data are also represented using Kaplan-Meier curves (**Figures 2C–J**).

### 3.4 Construction of nomogram

**Figure 3:**
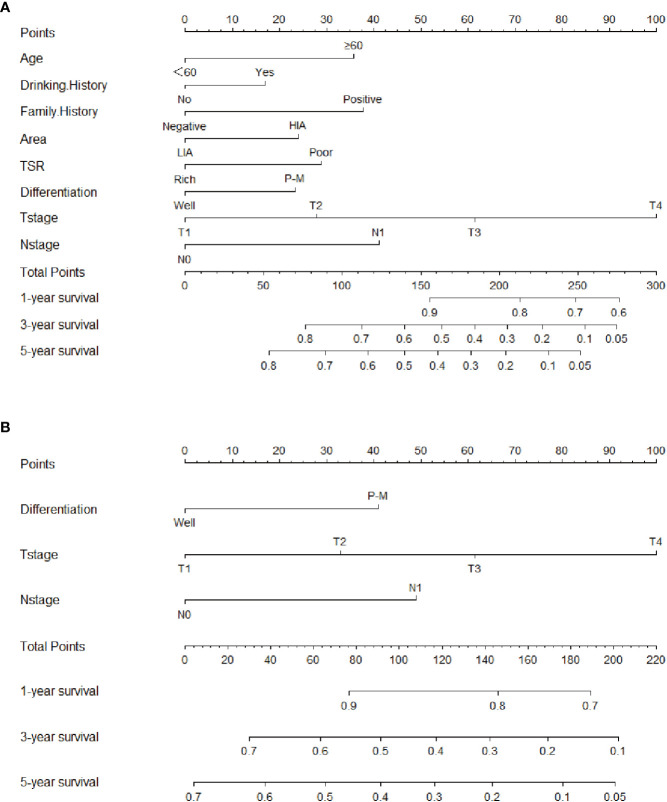
Clinical-pathological nomogram and TNM stage nomogram for 1-, 3-, and 5-year overall survival predicting in patients with esophageal squamous cell carcinoma. **(A)** Clinical-pathological nomogram. **(B)** TNM stage nomogram. HIA, High incidence area; LIA, Low incidence area; P-M, Poor or moderate differentiation.

To address the patient’s prognosis, we constructed a clinical-pathological nomogram (**Figure 3A**), and age, drinking history, family history, area, TSR, differentiation, T stage, and N stage were finally included into the model. Age was the age of patients at the time of diagnosis of the disease and was divided into two age groups, <60 and ≥60; drinking history was divided into positive and negative groups according to whether patients drank alcohol; family history was divided into positive and negative groups according to whether patients had tumor patients in their immediate family; area was divided into two groups, high and low incidence areas, according to the mortality rate of patients with ESCC in the area where patients lived (10); TSR was divided into stroma rich and stroma poor groups according to the results of this study; according to the AJCC stage, differentiation was divided into well differentiated, moderately differentiated, and poor differentiated, and T-stage was divided into T1, T2, T3 and T4; and N-stage was divided into N0 and N1. Among them, the differentiation was not an independent predictor for prognosis in the multivariate analysis, because it was included in the latest version of AJCC stage, differentiation was also included in the final nomogram. Since there was no difference in survival between moderate differentiation and poor differentiation patients in this study, thus combining them into one category. To investigate whether the clinical-pathological nomogram has incremental prognostic value for individualized OS prediction, a TNM stage nomogram model was also constructed, including only differentiation, T stage, N stage (**Figure 3B**). In the nomogram, each characteristic has its corresponding points, and total points is obtained by summing the scores corresponding to each characteristic. The scale at the bottom of the total points indicates the survival rates at 1, 3 and 5 years. The probability of survival at different periods can be obtained by looking at the probability of survival corresponding to the total points.

### 3.5 Calibration of clinical-pathological nomogram models

**Figure 4:**
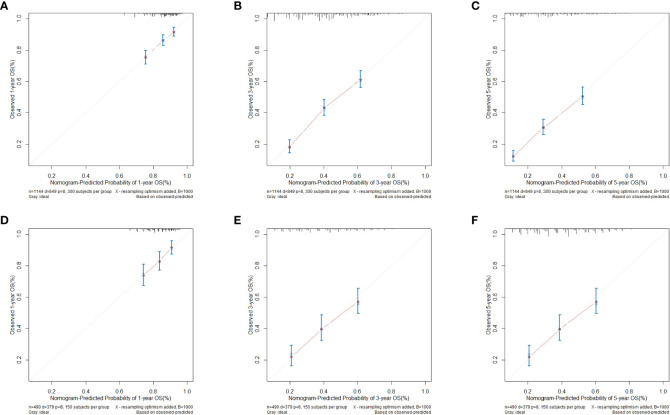
Calibration curves for clinical-pathological nomogram model in validation and training cohort. **(A)** 1-year calibration curve in training cohort. **(B)** 3-year calibration curve in the training cohort. **(C)** 5-year calibration curve in the training cohort. **(D)** 1-year calibration curve in the validation cohort. **(E)** 3-year calibration curve in the validation cohort. **(F)** 5-year calibration curve in the validation cohort.

**Figure 4** shows the calibration curve for the clinical-pathological nomogram model. The results show that the 1-, 3- and 5-year OS predictions of the clinical-pathological nomogram model well matched the actual outcomes in the training and validation cohort.

### 3.6 Validation of clinical-pathological nomogram model

The C-index of the clinical-pathological nomogram model and the TNM stage nomogram model in the training cohort were 0.643 (0.642-0.644) and 0.600 (0.599-0.601), respectively, and the clinical-pathological nomogram was better than the TNM stage in OS prediction. The 1-, 3- and 5-year NRI values of the clinical-pathological nomogram compared with TNM stage in the training cohort were 0.219 (0.039-0.433), 0.555 (0.396-0.677) and 0.491 (0.358-0.638), respectively, and the results showed that the prediction of the clinical-pathological nomogram was better than that of the traditional TNM stage in 1-, 3-, and 5-year OS prediction.

**Figure 5:**
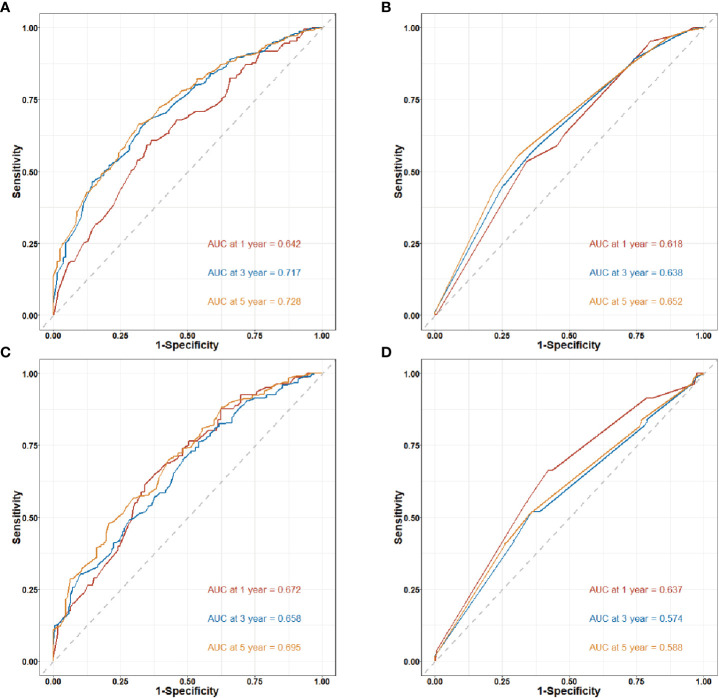
Time-dependent receiver operating characteristic (ROC) curves for nomogram in training and validation cohort. AUC. Area under the ROC curve; CI: confidence interval. **(A)** 1-year, 3-year and 5-year ROC curves of the clinical-pathological nomogram model in the training cohort. **(B)** 1-year, 3-year and 5-year ROC curves of the TNM stage nomogram model in the training cohort. **(C)** 1-year, 3-year and 5-year ROC curves of the clinical-pathological nomogram model in the validation cohort. **(D)** 1-year, 3-year and 5-year ROC curves of the TNM stage nomogram model in the validation cohort.

**Figure 5** shows the time-dependent ROC curves of the clinical-pathological nomogram and the TNM stage in the training and validation cohort. The results showed that in the training cohort, the area of under curve (AUC) of the 3 years and 5 years of the clinical-pathological nomogram model was significantly larger than that of the TNM stage nomogram model, while the AUC of the 1 year of the two models was similar, thus the clinical-pathological nomogram was better than the TNM stage at 3 years and 5 years (*P* < 0.001), and the two models were similar at 1 year (*P* = 0.414); in the validation cohort the results were similar to the training cohort, the clinical-pathological nomogram model was better than the TNM stage nomogram model at 3 years and 5 years (*P* < 0.001), and the two models were similar at 1 year (*P* = 0.306).

Clinical benefits of clinical-pathological nomogram model

**Figure 6:**
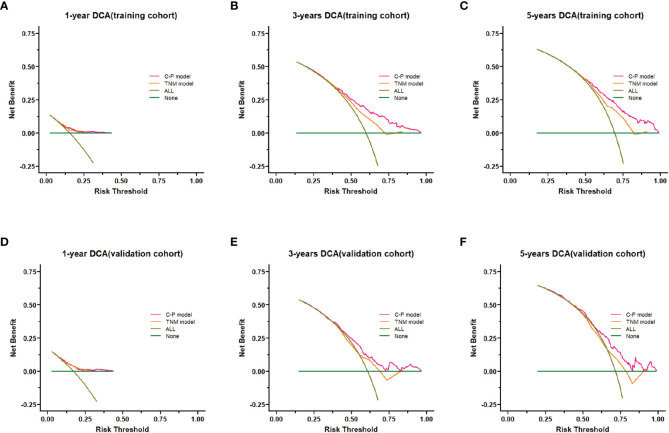
Decision curve analysis (DCA) of the nomogram model. **(A–C)** DCA curves of 1-,3- and 5-years survival prediction for the clinical-pathological nomogram model and the TNM stage nomogram model in the training cohort. **(D–F)** DCA curves of 1-,3- and 5-years survival predictions for the clinical-pathological nomogram model and the TNM stage nomogram model in the validation cohort. C-P model: clinical-pathological nomogram model.

The clinical benefit of the clinical-pathological nomogram model was compared with the TNM stage nomogram model. The Decision curve analysis (DCA) curve showed that when the threshold probability was > 0.5, the area under the decision curve of the clinical-pathological nomogram model was larger than that of the TNM stage nomogram model in the 3-year and 5-year, thus the clinical-pathological nomogram model could better predict the 3-year and 5-year OS of patients. And the clinical-pathological nomogram model yielded a larger net benefit than TNM stage nomogram model when the threshold probability was > 0.5 (**Figure 6**).

### 3.7 Performance of prognostic nomogram model

**Figure 7:**
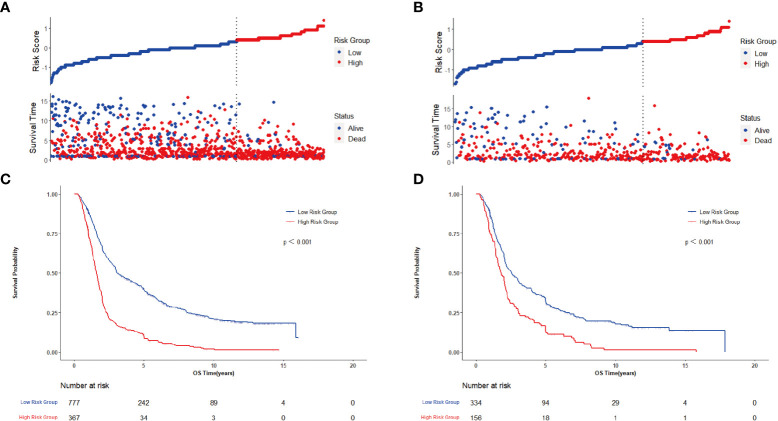
Evaluates of clinical-pathological nomogram model performance in training cohort and confirms on the basis of validation cohort. **(A)** Distribution of risk scores and survival status in training cohort; **(B)** Distribution of risk scores and survival status in validation cohort; **(C)** Kaplan-Meier survival curve in training cohort; **(D)** Kaplan-Meier survival curve in validation cohort.

**Figure 8:**
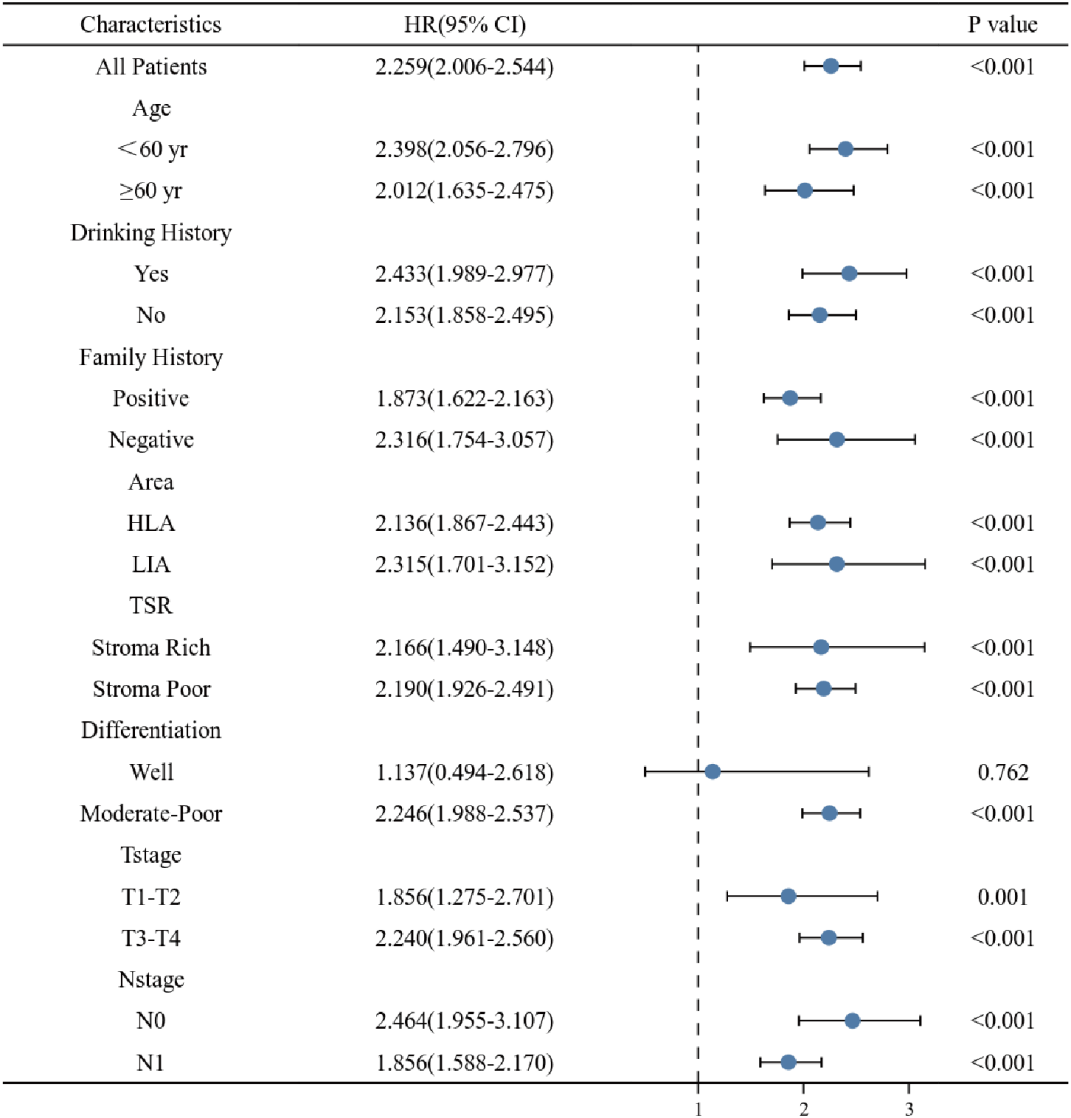
Stratified analysis of the clinical-pathological nomogram model in different subgroups.

For further revealing the prognostic prediction value of the clinical-pathological nomogram model, the X-tile software was used to categorized into high-risk and low-risk groups according to the optimal cut-off value of the total predictive scores in the training cohort. The relationships among risk scores and survival status of patients in training cohort and validation cohort are shown in **Figures 7A, B**. Kaplan-Meier survival curves revealed that patients with higher risk scores had significantly poorer OS (P<0.001, **Figures 7C, D**). Subgroup analyses were performed in the overall populations, and the clinical-pathological model also maintained a good and stable prediction performance (**Figure 8**).

## 4 Discussion

Notwithstanding the fact that great progress has been made in the prevention and treatment of ESCC in recent decades, the prognosis of ESCC is still poor with the high mortality rate (17, 18). The TNM stage system is adopted to predict the prognosis of numerous cancers, but TNM stage system is difficult to effectively predict the prognosis of individual patients due to multitudinous factors affecting the prognosis. For instance, patients with the same pathological stage, and treated by the same treatment plan, have completely dissimilar prognosis, indicating that there are biological differences between distinct patients. Furthermore, it is not enough to judge the prognosis on the basis of the TNM stage alone. As a consequence, there is a need to add new factors that affect survival and establish more reliable prognostic tools that can be used for risk stratification and further clinical decision-making, thereby which is advantageous for patients to ameliorate their prognosis.

Tumor is composed of cancerous cells and their surrounding complex stroma, stroma play a paramount role in tumor genesis and tumor metastasis (19). The occurrence and development of tumor is a complex process in which cancerous cells and stroma continuously interact and influence each other (20). There are complex stroma surrounding the tumor, including blood and lymphatic vessels, fibroblasts, endothelial cells, immune cells, cytokines, extra-cellular vesicles, and extra-cellular matrix (21). The extra-cellular matrix is the primary component of stroma. Apart from that, its function is not only limited to connect, support and protect cells, but also play a crucial role in cell proliferation and differentiation, cell migration, cell adhesion, angiogenesis, tissue development and repair (22–25). Aside from that, the accumulation and quantity of extra-cellular matrix components can result in tumor tissue fibrosis, which is required for wound healing and tissue repair to fight tissue infection, inflammation, autoimmune diseases, degenerative diseases, and tumors, resulting in prolonged survival (26). The stroma also contains a multitude of immune cells, such as NK cells, CD8+ cytotoxic T cells and antigen presenting cells (APCs), which can inhibit tumor growth and the immune response generated by the immune cells has anti-tumor properties (27). The stroma affects tumor growth and biological behavior. What’s more, the value of stroma in tumor prognosis prediction has been paid an heightening amount of attention (28). Tumor-stromal ratio (TSR) is a common stroma statistical method by calculation the ratio of area occupation of the stroma to tumor tissue. For the time being, a myriad of studies (29–31) have used the TSR in the prediction of postoperative prognosis of tumor patients. Furthermore, the results show that the TSR is independent predictor of prognosis in cancer patients. In the practice of Chinese doctors, those tumor with rich stroma are called hard cancers, and with poor stroma are called soft cancers. Aside from that, they found that the prognosis of hard cancers is better than that of soft cancers. In this investigation, we found a novel classification criteria by comparing the correlation between the richness of the stroma (tumor-stromal ratio) and survival in patients with ESCC. This classification criteria can be adopted to group patients by adopting TSR, which provides a basis for subsequent studies on the role of stroma in tumor development. In line with the classification criteria in this research, the stroma-rich group survived remarkably better than stroma-poor group. Apart from that, we also found that in the stromal rich group, the differentiation of patients was better than that in the stromal poor group, nevertheless, the depth of invasion was higher than that in the stromal poor group. With this classification criteria, the survival and clinical characteristics of patients in the two groups differed strikingly, which demonstrated that the classification criteria had great value. As a consequence, the TSR was incorporated into the construction of the prognostic model.

Esophageal cancer in China has the characteristics of conspicuous familial aggregation phenomenon and marked regional distribution differences (forming apparent high and low incidence areas) (1, 32). As already demonstrated by the epidemiological studies, individuals with a family history of esophageal cancer have a higher probability of developing esophageal cancer and a worse prognosis than individuals without a family history (33, 34). The reasons of higher risk of individuals with a positive family history may be on account of genetic inheritance. Apart from that, patients with a family history of esophageal cancer are more likely to have a genetic predisposition than those without a family history of cancer (34). Since family history is a reflection of an individual genetic risk factor, and it is believed that family history can be used as an indicator to be included in the predictive model for identification of high-risk groups (35). What’s more, the environment is also a pivotal factor affecting the occurrence of esophageal cancer. In China, there are striking geographical differences in the incidence of esophageal cancer, with pronounced high-incidence areas and low-incidence areas, the natural environment and living customs in diverse regions are crucial factors affecting the incidence of esophageal cancer (1, 32). As persuasively demonstrated by relevant studies (34, 36), environmental and genetic risk factors may exert collective influence on individual morbidity risk and prognosis survival. Shared genetic susceptibility and environmental exposure, or the possibility of their interaction, may be paramount factors in the development and progression of esophageal cancer. In this exploration, we found that patients with negative family history and patients in low-incidence areas had markedly better survival than patients with positive family history and patients in high-incidence areas (*P* < 0.05), so high and low incidence areas were incorporated into the model construction. Furthermore, age, drinking history, differentiation, T stage, and N stage are evidently prognostic factors that affect the survival of patients with esophageal cancer (37, 38). What’s more, they were also included in the construction of the prognostic model in this research.

In this investigation, we constructed a nomogram model to predict the prognosis of patients with ESCC. The nomogram has been extensively employed as a predictive method in oncology (39–41), it meets the requirements of an ensemble model and plays a role in driving personalized medicine for clinicians to use for prognosis prediction (42). Traditionally, tumor staging on the basis of the AJCC/UICC criteria has been the initial choice for predicting the prognosis of ESCC. Nevertheless, diverse prognoses were observed in patients at the same stage. This prognostic heterogeneity can be explained by age, family history, high and low incidence regions, and other factors that are not considered in tumor staging based upon AJCC/UICC criteria. This study included the largest sample size of patients with ESCC and the longest follow-up time to explored the association between TSR and clinical characteristics and prognosis of patients with ESCC, clarified that TSR is an crucial factor affecting the prognosis of patients with ESCC, and developed a novel criteria for the classification of TSR and include TSR in the nomogram model for patients with ESCC for the first time. A clinical-pathological nomogram model was constructed by combining the clinical information (age, drinking history, family history, and high-low risk area) and pathological information (TSR, differentiation, T stage and N stage) of patients. The C-index and NRI of the internally validated clinical-pathological model constructed in this exploration were remarkably higher than those of the TNM staging. In the training set and validation set, the 3-year and 5-year predictive ability of the clinical-pathological nomogram model was found to be strikingly better than that of the TNM staging (P < 0.001), indicating that the predictive performance of nomogram model constructed in this investigation was superior to that of TNM analysis. The results of decision curve analysis (DCA) indicated that the clinical-pathological model had a better net benefit than TNM staging in both the training and validation sets for 3-year and 5-year OS prediction. Based upon the risk scores of patients predicted by the nomogram model, patients were divided into two groups (high-risk and low-risk). Aside from that, the survival of patients in the high-risk group was conspicuously worse than that in the low-risk group (P < 0.001); in the subgroup analysis, the survival of patients in the high-risk group was noticeably worse than that in the low-risk group (P < 0.001). As evidently displayed by all these the research findings, the prognostic prediction model constructed in this research has better predictive and clinical application value than TNM staging.

There were some limitations to our research. First and foremost, our analysis may have been affected by bias owing to the retrospective of the study. Aside from that, in this exploration, whether the patients received chemoradiotherapy before surgery was considered, but may receive dissimilar treatments during the long postoperative course, but this factor was not addressed detailedly in our present study. Furthermore, this study used TMAs to observe TSR, which has the limitation of under-representation compared to whole sections.

## 5 Conclusion

This study confirmed that the TSR was an independent prognostic factor in patients with ESCC. Combined with tumor-stromal ratio and clinical and pathological factors, a prognostic nomogram is constructed, which has higher accuracy and better clinical value than traditional TNM stage. Furthermore, it provides a method for clinicians to predict ESCC prognosis and to adjust the treatment strategy.

## Data availability statement

The raw data supporting the conclusions of this article will be made available by the authors, without undue reservation.

## Ethics statement

The studies involving human participants were reviewed and approved by Ethical Committee of The First Affiliated Hospital of Zhengzhou University. The patients/participants provided their written informed consent to participate in this study.

## Author contributions

LDW, JFH and XS conceptualized and designed the study. FYZ, JLL, XZW, XML, RW, ZMF, XNH, BL, HJY, FBC, JLR and SLZ provided tumor samples collection and assembly. XKZ, RHX, LLL, YC, LYL JJJ, YZY and MMY provided patients’ information collection and follow-up. JFH, XS and KZ provided analyzed and interpreted the data. JFH, PPW and MXW drafted the manuscript. All authors reviewed the manuscript. All authors contributed to the article and approved the submitted version.
